# Supplementary material for: In vivo neutralization of coral snake venoms with an oligoclonal nanobody mixture in a murine challenge model
Source: Nat Commun. 2024 May 21;15:4310. doi: 10.1038/s41467-024-48539-z (PMC11109316; doi:10.1038/s41467-024-48539-z)
Supplement: Supplementary file 1 — Supplementary Information [file 41467_2024_48539_MOESM1_ESM.pdf]

Supplementary materials for

In vivo neutralization of coral snake venoms with an oligoclonal nanobody mixture

Melisa Benard-Valle[1], Yessica Wouters[1], Anne Ljungars[1], Giang Thi Tuyet Nguyen[1], Shirin Ahmadi[1], Tasja Wainani Ebersole[1], Camilla Holst Dahl[1], Alid Guadarrama-Martínez[2], Frederikke Jeppesen[1], Helena Eriksen[1], Gibran Rodríguez-Barrera[2], Kim Boddum[3], Timothy Patrick Jenkins[1], Sara Petersen Bjørn[1], Sanne Schoffelen[1], Bjørn Gunnar Voldborg[1], Alejandro Alagón[2], and Andreas Hougaard Laustsen[1]*

[1]Department of Biotechnology and Biomedicine, Technical University of Denmark, DK-2800 Kongens Lyngby, Denmark
[2]Departamento de Medicina Molecular y Bioprocesos, Instituto de Biotecnología, Universidad Nacional Autónoma de México, Avenida Universidad 2001, Cuernavaca, Mor., 62210, México.
[3]Sophion Bioscience, DK-2750 Ballerup, Denmark.

Corresponding author:
Andreas H. Laustsen
ahola@bio.dtu.dk

Supplementary Table 1. Immunization scheme per animal.

Day	Total venom dose (mg)	Dose per venom (mg)	Library generation
0	0.27	0.02	No
14	0.54	0.03	No
28	0.72	0.04	No
42	0.99	0.06	No
46	-	-	Yes
49	-	-	
56	1.26	0.07	No
70	1.44	0.08	No
84	1.80	0.10	No
98	2.16	0.12	No
102	-	-	Yes
105	-	-	

Supplementary Table 2. *In vivo* preincubation experiment with purified toxins

V_HH	Toxin	Assay	Molar ratio*	Toxin dose (µg/mouse)	V_HH dose (µg/mouse)	Total no of mice	No of dead mice	ToD 1	ToD 2	ToD 3
-	DH	**Toxin only (*i.v.*) control**	-	6 µg ($3LD_{50}$s)	0	3	3	10 min	10 min	10 min
TPL0629_01_D11	DH	Preincubation	2.5	6	31	3	0	**>24 h**	**>24 h**	**>24 h**
TPL0629_01_D11	DH	Preincubation	1	6	12.4	3	0	**>24 h**	**>24 h**	**>24 h**
TPL0629_01_G06	DH	Preincubation	2.5	6	31	3	0	120 min	120 min	120 min
TPL0629_01_A07	DH	Preincubation	2.5	6	30.2	3	0	**>24 h**	**>24 h**	**>24 h**
-	PLA_2N	**Toxin only (*i.v.*) control**	-	30.8 µg ($3LD_{50}$s)	0	3	3	45 min	60 min	80 min
TPL0637_01_A01	PLA_2N	Preincubation	5	30.8	192.8	3	0	**>24 h**	**>24 h**	**>24 h**
TPL0637_01_A01	PLA_2N	Preincubation	1	30.8	38.6	3	0	**>24 h**	**>24 h**	**>24 h**
TPL0637_01_A07	PLA_2N	Preincubation	5	30.8	210.9	3	0	**>24 h**	**>24 h**	**>24 h**
TPL0637_01_A07	PLA_2N	Preincubation	2.5	30.8	105.4	3	0	**>24 h**	**>24 h**	**>24 h**
TPL0637_01_A07	PLA_2N	Preincubation	1	30.8	42.2	3	0	**>24 h**	**>24 h**	**>24 h**
TPL0638_01_C09	PLA_2N	Preincubation	5	30.8	212.5	3	0	**>24 h**	**>24 h**	**>24 h**
TPL0638_01_C09	PLA_2N	Preincubation	1	30.8	42.5	3	0	**>24 h**	**>24 h**	**>24 h**
TPL0629_01_D11	PLA_2N	**Isotype control**	5	30.8	189.1	3	3	60 min	60 min	60 min
TPL0637_01_A07	-	**V_HH only control (*i.v.*)**	-	0	178.6	3	0	**>24 h**	**>24 h**	**>24 h**
TPL0629_01_D11	-	**V_HH only control (*i.v.*)**	-	0	31	3	0	**>24 h**	**>24 h**	**>24 h**

ToD. Time of death

*Number of V_HH molecules per toxin molecule.

Toxins and V_HHs were preincubated 30 min at 37 °C and then injected *i.v.*

Supplementary Table 3. *In vivo* rescue experiment with purified toxins.

V_HH	Toxin	Assay	Molar ratio*	Toxin Dose (*s.c.*) (µg/mouse)	V_HH dose (*i.v.*) (µg/mouse)	Total no. of mice	No. of dead mice	ToD 1	ToD 2	ToD 3
-	DH	**Toxin only (*s.c.*) control**	-	14.4 µg (3LD_{50})	-	3	3	20 min	24 min	28 min
**TPL0629_01_A07**	DH	Rescue	2.5	14.4	74.4	3	2	10 h	10 h	**>24 h**
**TPL0629_01_D11**	DH	Rescue	2.5	14.4	74.4	3	3	10 h	10 h	10 h
**TPL0629_01_D11**	DH	Rescue	5	14.4	148.9	3	2	18 h	18 h	**>24 h**
**TPL0629_01_D11**	DH	Rescue	10	14.4	297.8	3	0	**>24 h**	**>24 h**	**>24 h**
**TPL0629_01_D11 bivalent**	DH	Rescue	1.25	14.4	68.1	3	3	1.7 h	1.7 h	1.7 h
**TPL0629_01_D11 bivalent**	DH	Rescue	2.5	14.4	136.2	2	2	2 h	2 h	-
**TPL0629_01_D11 V_HH-Fc**	DH	Rescue	1.25	14.4	174.0	2	2	6.5 h	8.5 h	-
-	PLA_2N	**Toxin only (*s.c.*) control**	-	103.9 µg (3LD_{50})	-	3	3	60 min	60 min	80 min
**TPL0637_01_A01**	PLA_2N	Rescue	2.5	103.9	325.4	3	2	3 h	3 h	**>24 h**
**TPL0638_01_C09**	PLA_2N	Rescue	2.5	103.9	358.6	3	2	12 h	20 h	**>24 h**
**TPL0637_01_A07**	PLA_2N	Rescue	2.5	103.9	355.9	3	0	**>24 h**	**>24 h**	**>24 h**

* Number of V_HH molecules per toxin molecule.

ToD. Time of death

Toxins were injected *s.c.* and V_HHs were immediately afterwards injected *i.v.*

Supplementary Table 4. Design of oligoclonal mixtures.

	**V_HH Mix 1**		**V_HH Mix 2**	
**Venom**	*M. fulvius*		*M. diastema*	
**LD_{50} (µg/mouse)**	5.7		6.0	
**LD_{50} x 3**	17.1		18.0	
**Toxin family**	3FTx	PLA_2	3FTx	PLA_2
**Abundance in venom (%)**	32	60	22	62
**Approx. MW (Da)**	6 500	14 000	6 500	14 000
**nmol of toxin in $3LD_{50}$s**	0.84	0.73	0.6	0.8
**Molar Ratio (Tx:V_HH)**	1 : 10	1 : 10	1 : 10	1 : 10
**V_HH**	**TPL0629_01_D11**	**TPL0637_01_A07**	**TPL0629_01_D11**	**TPL0637_01_A07**
**nmol of V_HH**	8.4	7.3	6.09	7.97
**MW (Da)**	16 500	18 400	16 500	18 400
**µg of V_HH**	139.0	134.9	100.6	146.8

Supplementary Table 5. *In vivo* preincubation experiment with whole coral snake venoms

Treatment	Venom	Assay	Toxin abundance in venom*	Molar ratio**	Venom Dose (µg/mouse)	V_HH Dose (µg/mouse)	Total no. of mice	No of dead mice	ToD 1	ToD 2	ToD 3
-	*M. fulvius*	**Venom only (*i.v.*) control**		-	17.1 ($3LD_{50}$)	0	3	3	1.5 h	1.5 h	1.5 h
**TPL0629_01_D11**<br>**TPL0637_01_A01**	*M. fulvius*	Preincubation	3FTxs 22%<br>PLA_2s 60%	2<br>2	17.1<br>17.1	19.1<br>24.7	3	3	70 m	70 m	70 m
**TPL0629_01_D11**<br>**TPL0637_01_A07**	*M. fulvius*	Preincubation	3FTxs 22%<br>PLA_2s 60%	2<br>2	17.1<br>17.1	19.1<br>27.0	3	2	2 h	13 h	**>24 h**
**TPL0629_01_D11**<br>**TPL0637_01_A07**	*M. fulvius*	Preincubation	3FTxs 22%<br>PLA_2s 60%	4<br>4	17.1<br>17.1	38.2<br>54.0	3	2	12 h	12 h	**>24 h**
**TPL0629_01_D11**<br>**TPL0637_01_A07**	*M. fulvius*	Preincubation	3FTxs 40%<br>PLA_2s 60%	4<br>4	17.1<br>17.1	69.5<br>54.0	3	2	14 h	17.5 h	**>24 h**
**TPL0629_01_D11**<br>**TPL0637_01_A07**	*M. fulvius*	Preincubation	3FTxs 32%<br>PLA_2s 60%	8<br>8	17.1<br>17.1	111.2<br>107.9	3	3	3 h	3 h	3 h
**TPL0629_01_D11**<br>**TPL0637_01_A07**	*M. fulvius*	Preincubation	3FTxs 32%<br>PLA_2s 60%	8<br>8	17.1<br>17.1	111.2<br>107.9	3	3	3 h	3 h	3 h
**TPL0629_01_D11**<br>**TPL0637_01_A07**	*M. fulvius*	Preincubation	3FTxs 32%<br>PLA_2s 60%	10<br>10	17.1<br>17.1	139.0<br>134.9	3	0	**>24 h**	**>24 h**	**>24 h**
**Coralmyn**	*M. fulvius*	Preincubation	3FTxs 32%<br>PLA_2s 60%	5[#]<br>5[#]	17.1<br>17.1	463.0<br>403.1	3	0	**>24 h**	**>24 h**	**>24 h**
**TPL0637_01_A07**	*M. fulvius*	Preincubation	PLA_2s 60%	8	17.1	107.9	3	3	3 h	3 h	3 h
**TPL0637_01_A07**	*M. fulvius*	Preincubation	PLA_2s 60%	10	17.1	134.9	3	3	2.5 h	3.5 h	3.5 h
**TPL0629_01_D11**	*M. fulvius*	Preincubation	3FTxs 32%	10	17.1	139.0	3	3	1.5 h	1.5 h	1.5 h
-	*M. diastema*	**Venom only (*i.v.*) control**		-	18 ($3LD_{50}$)	0	3	3	1.5 h	1.5 h	1.5 h
**TPL0629_01_D11**<br>**TPL0637_01_A01**	*M. diastema*	Preincubation	3FTxs 22%<br>PLA_2s 60%	2<br>2	18<br>18	20.1<br>26.0	3	3	10 h	10 h	10 h
**TPL0629_01_D11**<br>**TPL0637_01_A07**	*M. diastema*	Preincubation	3FTxs 22%<br>PLA_2s 60%	8<br>8	18<br>18	80.5<br>117.4	3	3	3 h	3 h	4.5 h
**TPL0629_01_D11**<br>**TPL0637_01_A07**	*M. diastema*	Preincubation	3FTxs 22%<br>PLA_2s 62%	10<br>10	18<br>18	100.6<br>146.8	3	1	10 h	**>24 h**	**>24 h**
**Coralmyn**	*M. diastema*	Preincubation	3FTxs 22%<br>PLA_2s 62%	5[#]<br>5[#]	18<br>18	335.1<br>438.4	3	3	21 m	23 m	1.7 h
**TPL0637_01_A07**	*M. diastema*	Preincubation	PLA_2s 62%	10	18	146.8	3	3	2.5 h	2.5 h	2.5 h
**TPL0637_01_A07**	*M. diastema*	Preincubation	PLA_2s 60%	8	18	113.6	3	3	3 h	3 h	3 h
**TPL0629_01_D11**	*M. diastema*	Preincubation	3FTxs 22%	10	18	100.6	3	3	2.5 h	1.5 h	1.5 h

*Percentage of total venom considered to be composed of a specific toxin family, based on proteomic data. Toxin abundance considered was empiricaly modified in some cases to attempt an improvement in neutralization.

**Number of V_HH molecules per toxin molecule in venom. Only PLA_2s and 3FTxs were considered.

[#] Equivalent to a 1 in 10 toxin to binding sites ratio

ToD. Time of death

A

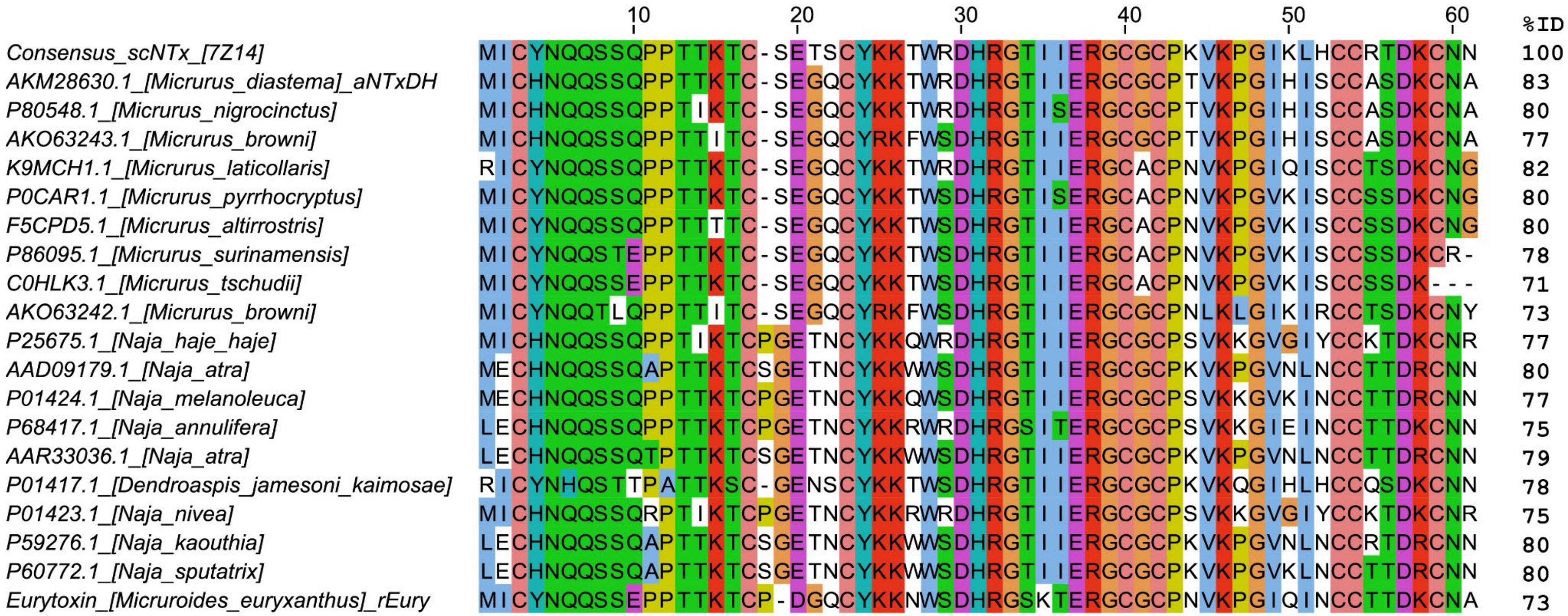
10 20 30 40 50 60
%ID
Consensus_scNTx_[7Z14] MICYNQQSSQPPTTKTC-SETSCYKKTWRDHRGTIIERGCGCPKVKPGIKLHCCRTDKCNN 100
AKM28630.1_[Micrurus_diastema]_aNTxDH MICHNQQSSQPPTTKTC-SEGQCYKKTWRDHRGTIIERGCGCPTVKPGIHISCCASDKCNA 83
P80548.1_[Micrurus_nigrocinctus] MICHNQQSSQPPTIKTC-SEGQCYKKTWRDHRGTISERGCGCPTVKPGIHISCCASDKCNA 80
AKO63243.1_[Micrurus_browni] MICHNQQSSQPPTTITC-SEGQCYRKFWSDHRGTIIERGCGCPTVKPGIHISCCASDKCNA 77
K9MCH1.1_[Micrurus_laticollaris] RICYNQQSSQPPTTKTC-SEGQCYKKTWRDHRGTIIERGCACPNVKPGIQISCCTSDKCNG 82
P0CAR1.1_[Micrurus_pyrrhocryptus] MICYNQQSSQPPTTKTC-SEGQCYKKTWSDHRGTISERGCACPNVKPGVKISCCSSDKCNG 80
F5CPD5.1_[Micrurus_altirrostris] MICYNQQSSQPPTTTTC-SEGQCYKKTWSDHRGTIIERGCACPNVKPGVKISCCSSDKCNG 80
P86095.1_[Micrurus_surinamensis] MICYNQQSTEPPTTKTC-SEGQCYKKTWSDHRGTIIERGCACPNVKPGVKISCCSSDKCR- 78
C0HLK3.1_[Micrurus_tschudii] MICYNQQSSEPPTTKTC-SEGQCYKKTWSDHRGTIIERGCACPNVKPGVKISCCSSDK--- 71
AKO63242.1_[Micrurus_browni] MICYNQQTLQPPTTITC-SEGQCYRKFWSDHRGTIIERGCGCPNLKLGIKIRCCTSDKCNY 73
P25675.1_[Naja_haje_haje] MICHNQQSSQPPTIKTCPGETNCYKKQWRDHRGTIIERGCGCPSVKKGVGIYCCKTDKCNR 77
AAD09179.1_[Naja_atra] MECHNQQSSQAPTTKTCSGETNCYKKWWSDHRGTIIERGCGCPKVKPGVNLNCCTTDRCNN 80
P01424.1_[Naja_melanoleuca] MECHNQQSSQPPTTKTCPGETNCYKKQWSDHRGTIIERGCGCPSVKKGVKINCCTTDRCNN 77
P68417.1_[Naja_annulifera] LECHNQQSSQPPTTKTCPGETNCYKKRWRDHRGSITERGCGCPSVKKGIEINCCTTDKCNN 75
AAR33036.1_[Naja_atra] LECHNQQSSQTPTTKTCSGETNCYKKWWSDHRGTIIERGCGCPKVKPGVNLNCCTTDRCNN 79
P01417.1_[Dendroaspis_jamesoni_kaimosae] RICYNHQSTTPATTKSC-GENSCYKKTWSDHRGTIIERGCGCPKVKQGIHLHCCQSDKCNN 78
P01423.1_[Naja_nivea] MICHNQQSSQRPTIKTCPGETNCYKKRWRDHRGTIIERGCGCPSVKKGVGIYCCKTDKCNR 75
P59276.1_[Naja_kaouthia] LECHNQQSSQAPTTKTCSGETNCYKKWWSDHRGTIIERGCGCPKVKPGVNLNCCRTDRCNN 80
P60772.1_[Naja_sputatrix] LECHNQQSSQAPTTKTCSGETNCYKKWWSDHRGTIIERGCGCPKVKPGVKLNCCTTDRCNN 80
Eurytoxin_[Micruroides_euryxanthus]_rEury MICYNQQSSEPPTTKTCP-DGQCYKKNWSDHRGSKTERGCGCPNVKPGIQINCCTTDKCNA 73

B

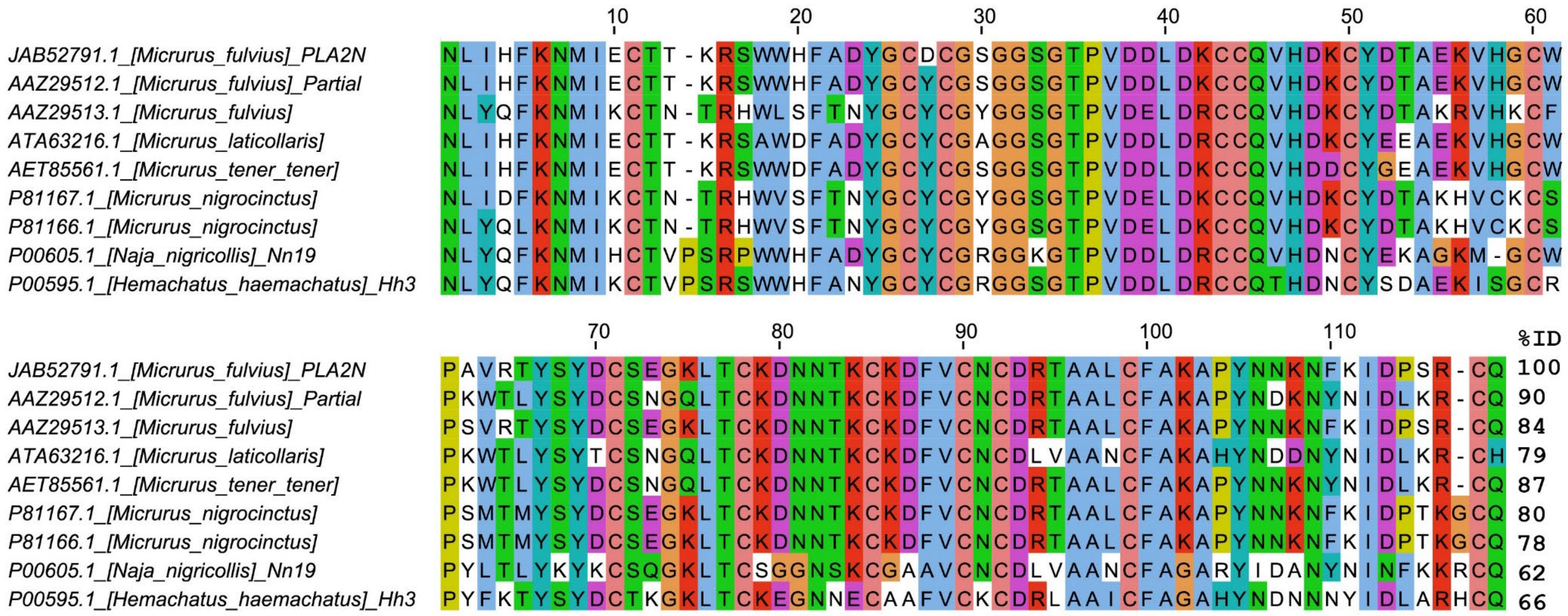

Supplementary Figure 1. Multiple sequence alignment of selected short chain αNTxs and PLA_2s from elapid snake venoms. **A.** Sequence alignment of short chain αNtx sequences including the ones used in this work and other similar ones from *Micrurus* (coral snakes), *Naja* (cobras), and *Dendroaspi*s (mambas) snake venoms. **B.** Sequence alignment of PLA_2 sequences including PLA_2N (used in this work) and other similar ones from *Micrurus*, *Naja,* and *Hemachatus* (rinkhals) snake venoms. The analysis was performed using Jalview v2.11.3.2. The signal peptide sequences were excluded, and only mature peptide sequences were considered. %ID: Percentage sequence identity to scNTx (A) or PLA_2N (B).

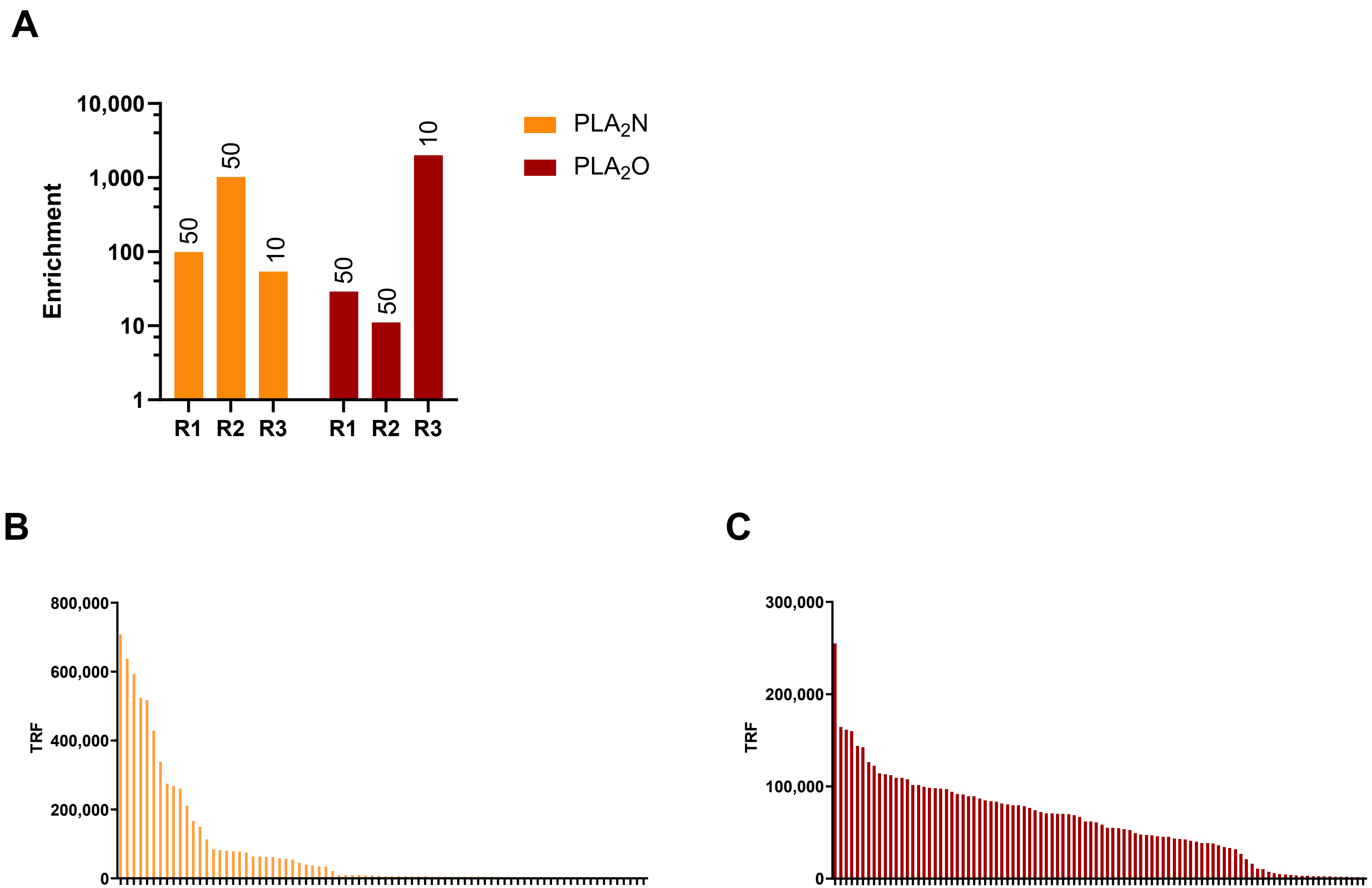

Supplementary Figure 2. Enrichment of phages in the selection campaigns and primary screening of monoclonal V_HHs against PLA_2s. **A.** Enrichment of phages in the selection campaigns on PLA_2N and PLA_2O. The consecutive selection rounds are labeled R1 to R3. The enrichment is calculated as the phage titer obtained after selection on the antigen divided with a titer of a control selection run in parallel without antigen. The antigen concentration (nM) in each round is shown over the bars. **B-C.** Primary screening of monoclonal V_HHs in an expression normalized DELFIA testing the binding to various toxins. Each bar represents an individual clone. TRF. Time Resolved Fluorescence. **B.** Binding of V_HHs from a selection campaign on PLA_2N to PLA_2N. **C.** Binding of V_HHs from a selection campaign on PLA_2O to PLA_2O.

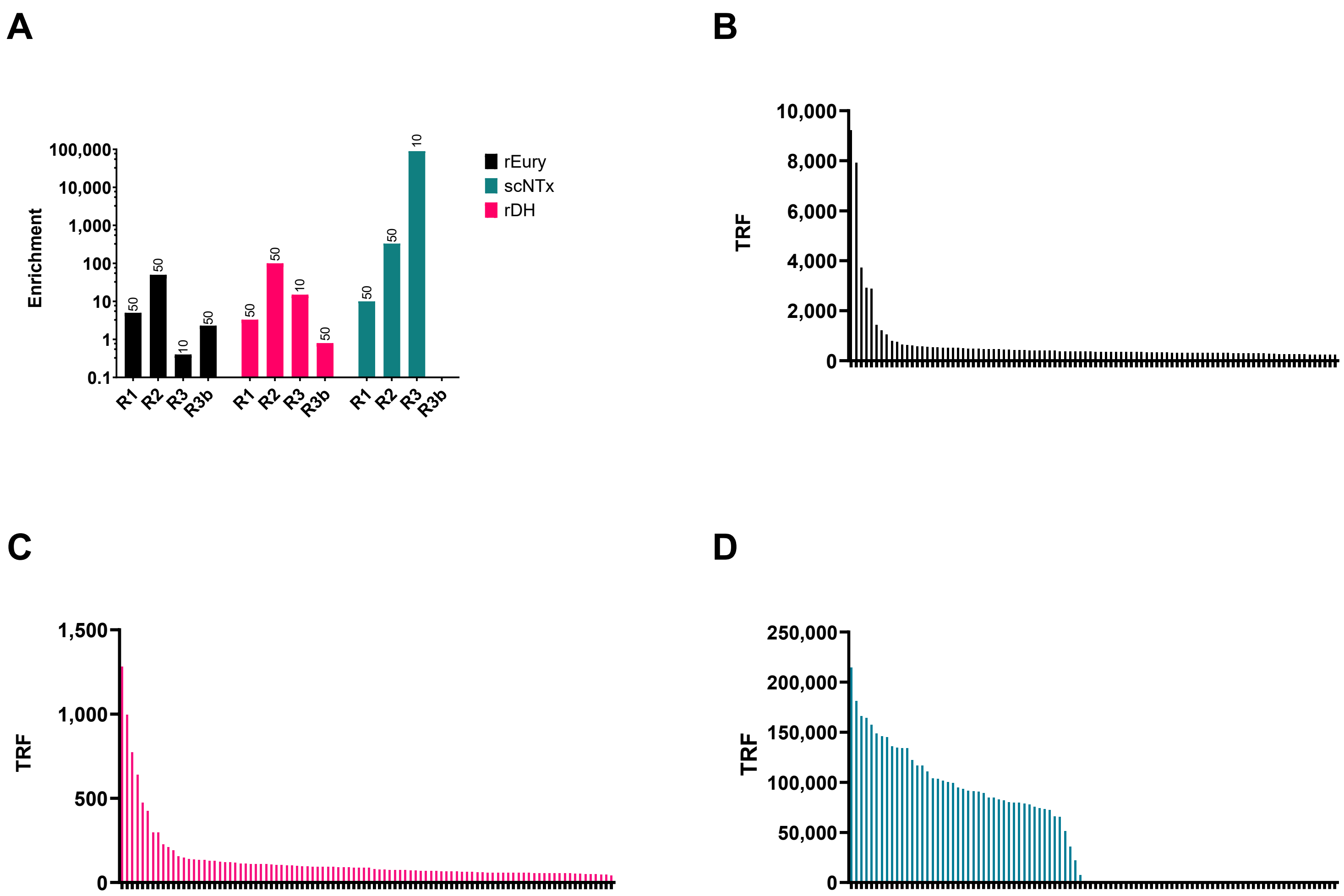

Supplementary Figure 3. Enrichment of phages in the selection campaigns and primary screening of monoclonal V_HHs against aNTxs. **A.** Enrichment of phages in the selection campaigns on rEury, rDH, and scNTx. The consecutive selection rounds are labeled R1 to R3b. The enrichment is calculated as the phage titer obtained after selection on the antigen divided with the titer of a control selection run in parallel without antigen. The antigen concentration (nM) in each round is shown over the bars. **B-D.** Primary screening of monoclonal V_HHs in an expression normalized DELFIA testing the binding to various toxins. Each bar represents an individual clone. TRF. Time Resolved Fluorescence. **B.** Binding of V_HHs from a selection campaign on rEury to rEury. **C.** Binding of V_HHs from a selection campaign on rDH to rDH. **D.** Binding of V_HHs from a selection campaign on the consensus toxin scNTx to scNTx.

A. Anti-αNTx V_HH sequences

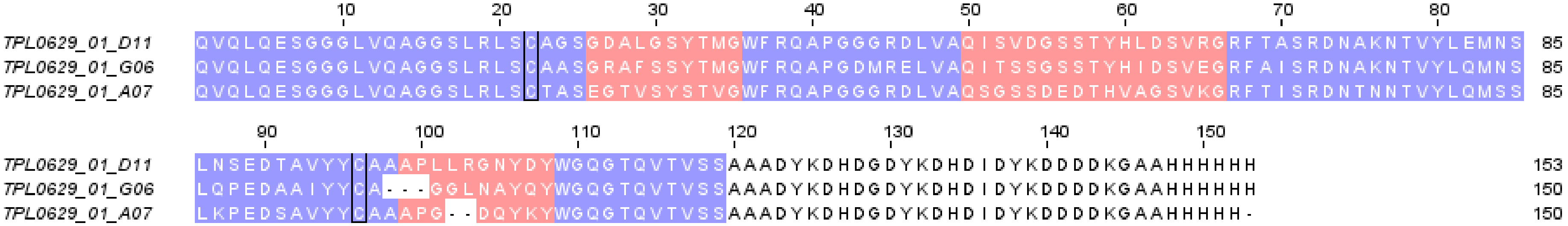

B. Anti-PLA_2 V_HH sequences

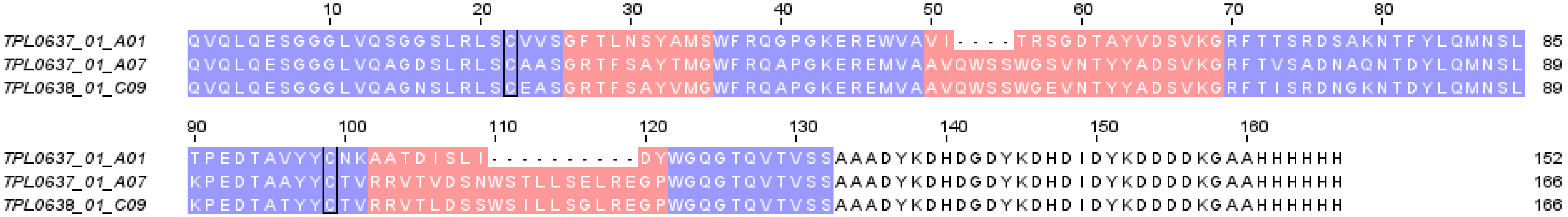

Supplementary Figure 4. Amino acid sequence alignment of V_HHs selected for *in vitro* and *in vivo* neutralization. **A.** Sequences of αNTx-targeting V_HHs. **B.** Sequences of PLA_2-targeting V_HHs. Framework regions are labeled purple and CDR regions pink. Cysteine residues are marked inside black boxes. The 3xFLAG and 6x His tags in the end are left unlabeled. The alignment was performed using Jalview v2.11.3.2.

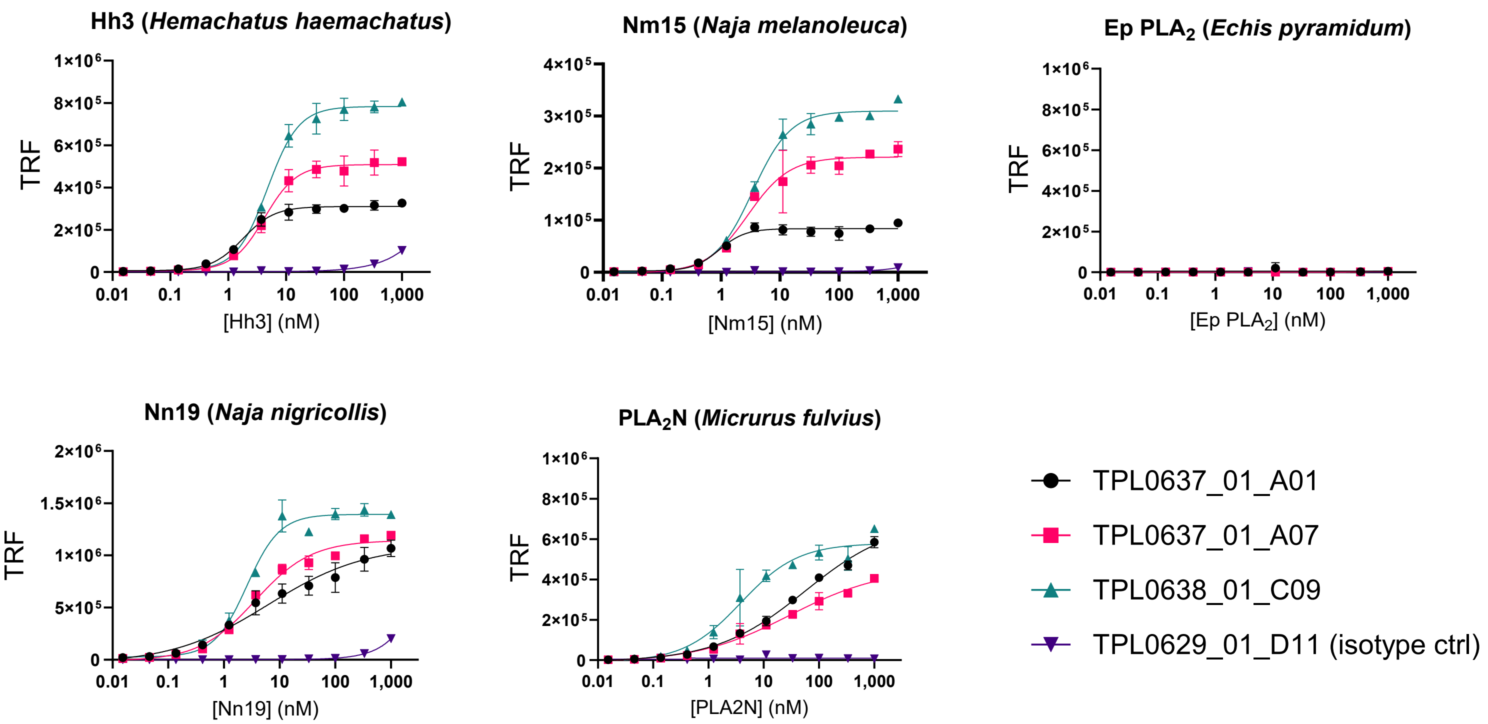

	Hh3		Nm15		Nn19		PLA_2N		Ep PLA_2	
	EC_{50} (nM)	R^2	EC_{50} (nM)	R^2	EC_{50} (nM)	R^2	EC_{50} (nM)	R^2	EC_{50} (nM)	R^2
**TPL0637_01_A01**	1.9	0.988	1.0	0.967	5.1	0.964	43.2	0.994	ND	ND
**TPL0637_01_A07**	4.5	0.986	2.9	0.972	3.6	0.990	28.0	0.982	2.6	0.596
**TPL0638_01_C09**	5.1	0.995	3.6	0.993	2.7	0.986	4.5	0.962	2.8	0.721
**TPL0629_01_D11 (isotype)**	>1000	0.992	>1000	0.718	>1000	0.990	ND	ND	ND	ND

Supplementary Figure 5. Cross-reactivity of anti-PLA_2 V_HHs determined by a capture DELFIA. Purified PLA_2s or PLA_2 containing fractions from diverse Elapid snake species were diluted from 1000 to 0.01 nM and left to bind the individual V_HHs. As a negative isotype control, the anti-αNTx V_HH TPL0629_01_D11 was included, and as a negative control antigen, a PLA_2 from the venom of *Echis pyramidum* was included. The table shows the EC_{50} values in nM for the V_HHs binding to the various PLA_2s. The values were obtained from a sigmoidal curve-fit in the graphs. *R^2*. R squared expressing the goodness-of-fit for the non-linear regressions. *ND.* not determined, as no binding was observed.

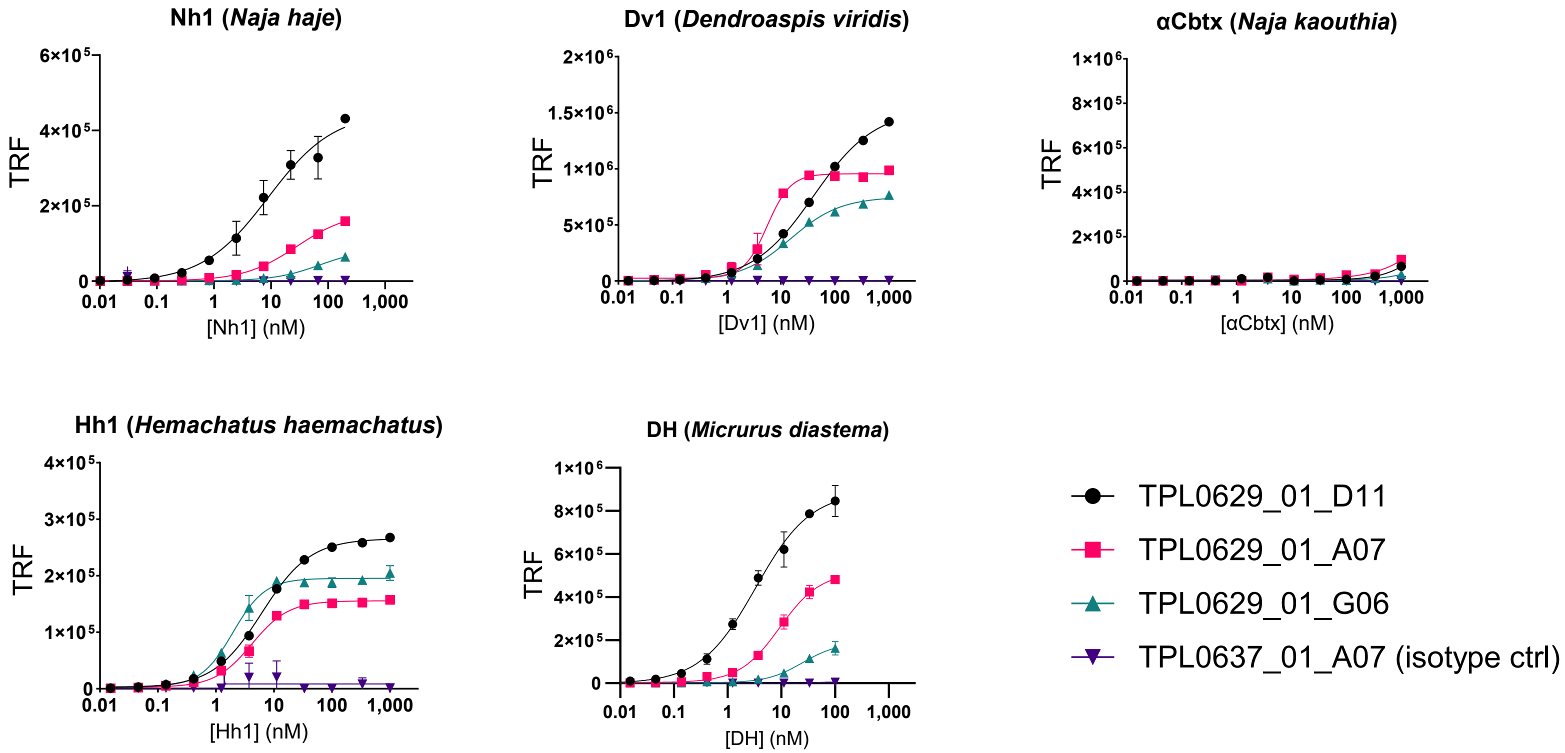

	Nh1		Dv1		Hh1		DH		aCbtx	
	EC_{50} (nM)	R^2	EC_{50} (nM)	R^2	EC_{50} (nM)	R^2	EC_{50} (nM)	R^2	EC_{50} (nM)	R^2
**TPL0629_01_D11**	8.0	0.974	37.9	0.999	6.6	0.999	3.1	0.992	>1000	0.894
**TPL629_01_A07**	24.1	0.997	5.8	0.991	4.4	0.995	9.1	0.995	>1000	0.916
**TPL0629_01_G06**	60.9	0.993	15.9	0.996	2.1	0.993	23.7	0.983	>1000	0.835
**TPL0637_01_A07 (isotype)**	ND	ND	363.3	0.200	1.4	0.120	62.9	0.633	ND	ND

Supplementary Figure 6. Cross-reactivity of anti-αNTx V_HHs determined by a capture DELFIA. Different concentrations of recombinantly produced αNTx or αNTx containing fractions from diverse elapid snake species were diluted from 1000 to 0.01 nM and left to bind the individual V_HHs. As a negative isotype control, the anti-PLA_2 V_HH TPL0637_01_A07 was included and as a negative control antigen the long chain αNtx from the venom of *Naja kaouthia*, αCbtx, was included. The table shows the EC_{50} values in nM for the V_HHs binding to the various PLA_2s. The values were obtained from a sigmoidal curve-fit in the graphs. *R^2*. R squared expressing the goodness-of-fit for the non-linear regressions. *ND.* not determined, as no binding was observed.

A

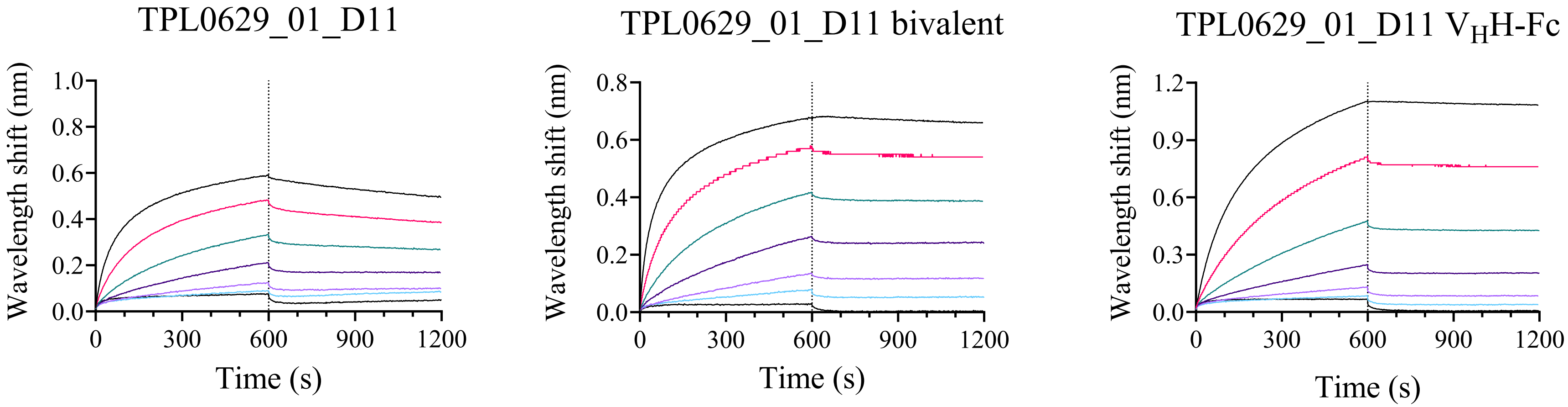

B

VHH	KD (M)	KD error	kon (1/Ms)	kon error	kdis (1/s)	kdis error	R^2
TPL0629_01_D11	$6.94 \cdot 10^{-09}$	$5.92 \cdot 10^{-11}$	$7.47 \cdot 10^{+04}$	$4.41 \cdot 10^{+02}$	$5.18 \cdot 10^{-04}$	$3.19 \cdot 10^{-06}$	0.995
TPL0629_01_D11 bivalent	$5.25 \cdot 10^{-10}$	$5.18 \cdot 10^{-11}$	$9.06 \cdot 10^{+04}$	$7.88 \cdot 10^{+02}$	$4.76 \cdot 10^{-05}$	$4.67 \cdot 10^{-06}$	0.983
TPL0629_01_D11 VHH-Fc	$3.69 \cdot 10^{-09}$	$1.51 \cdot 10^{-10}$	$2.92 \cdot 10^{+04}$	$2.34 \cdot 10^{+02}$	$1.08 \cdot 10^{-04}$	$4.33 \cdot 10^{-06}$	0.990

Supplementary Figure 7. Binding kinetics of TPL0629_01_D11 V_HH as monovalent and bivalent constructs. A. BLI sensorgrams showing the binding of TPL0629_01_D11 as a monovalent V_HH, a bivalent V_HH construct, and V_HH-Fc construct, to αNTx DH. **B.** BLI binding parameters.

A

Fraction	Toxin Family	Ret. Time	Abundance*	Fraction	Toxin Family	Ret. Time	Abundance*
A	ND	11.12	1.3	J	ND	36.37	2.2
B	ND	12.50	1.7	K	PLA_2	37.50	3.2
U	ND	13.30	0.4	L	PLA_2	38.32	2.7
V	3FTx	28.12	2.5	M	PLA_2	38.90	8.7
C	3FTx	29.76	2.1	N	PLA_2	39.69	18.1
D	3FTx	30.37	2.9	O	PLA_2	40.22	15.3
E	3FTx	32.22	5.2	P	3FTx	41.14	7.0
F	3FTx	33.90	4.6	Q	ND	44.27	0.4
G	3FTx	34.35	4.0	R	PLA_2	44.96	1.5
H	3FTx	34.60	3.6	S	ND	49.07	2.9
I	PLA_2	35.92	8.8	T	ND	51.50	1.0

*Percentage of the toxin in venom, based on area under curve from RP-HPLC chromatogram (A214 nm)

B

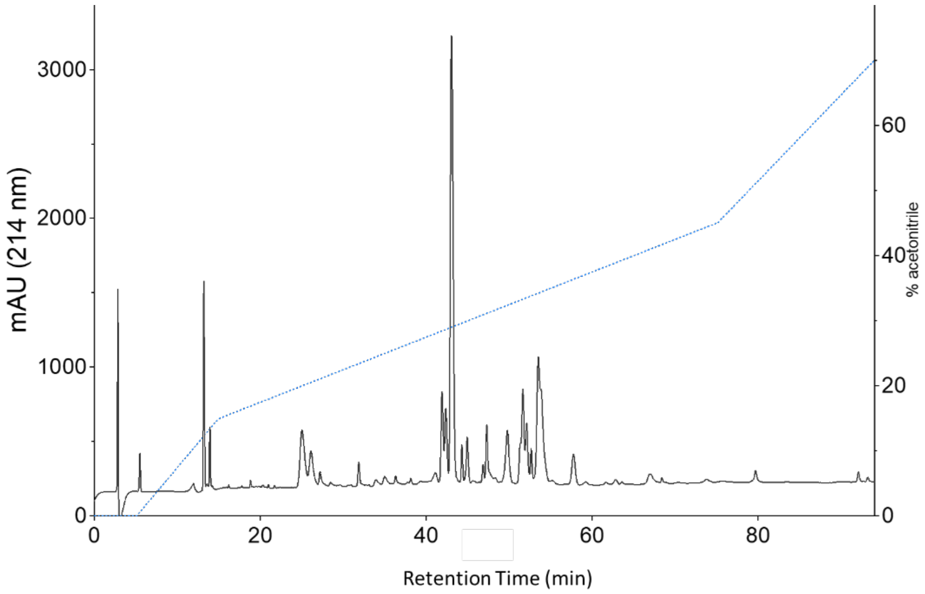

Supplementary Figure 8. Protein composition of the two coral snake venoms used in this work. A. RP-HPLC fraction identity and abundance in the venom of *Micrurus fulvius*. Modified from Vergara *et al.*, 2014. **B.** RP-HPLC chromatogram of the venom of *Micrurus diastema*.
