# Supplementary material for: In vivo neutralization of coral snake venoms with an oligoclonal nanobody mixture in a murine challenge model
Source: Nat Commun. 2024 May 21;15:4310. doi: 10.1038/s41467-024-48539-z (PMC11109316; doi:10.1038/s41467-024-48539-z)
Supplement: Supplementary file 3 — Reporting Summary [file 41467_2024_48539_MOESM3_ESM.pdf]

nature portfolio

Corresponding author(s): Andreas H. Laustsen

Last updated by author(s): Apr 12, 2024

Reporting Summary

Nature Portfolio wishes to improve the reproducibility of the work that we publish. This form provides structure for consistency and transparency in reporting. For further information on Nature Portfolio policies, see our Editorial Policies and the Editorial Policy Checklist.

Statistics

For all statistical analyses, confirm that the following items are present in the figure legend, table legend, main text, or Methods section.

n/a	Confirmed	
☐	☒	The exact sample size (n) for each experimental group/condition, given as a discrete number and unit of measurement
☒	☐	A statement on whether measurements were taken from distinct samples or whether the same sample was measured repeatedly
☐	☒	The statistical test(s) used AND whether they are one- or two-sided<br>*Only common tests should be described solely by name; describe more complex techniques in the Methods section.*
☒	☐	A description of all covariates tested
☐	☒	A description of any assumptions or corrections, such as tests of normality and adjustment for multiple comparisons
☐	☒	A full description of the statistical parameters including central tendency (e.g. means) or other basic estimates (e.g. regression coefficient) AND variation (e.g. standard deviation) or associated estimates of uncertainty (e.g. confidence intervals)
☐	☒	For null hypothesis testing, the test statistic (e.g. F, t, r) with confidence intervals, effect sizes, degrees of freedom and P value noted<br>*Give P values as exact values whenever suitable.*
☒	☐	For Bayesian analysis, information on the choice of priors and Markov chain Monte Carlo settings
☒	☐	For hierarchical and complex designs, identification of the appropriate level for tests and full reporting of outcomes
☒	☐	Estimates of effect sizes (e.g. Cohen's d, Pearson's r), indicating how they were calculated

Our web collection on statistics for biologists contains articles on many of the points above.

Software and code

Policy information about availability of computer code

Data collection	Patch Clamp data was collected with Sophion Analyzer v6.6.70 (Sophion Bioscience). BLI data was collected using Octet® Analysis Studio 12.2.2.26 (ForteBio). DELFIA data was collected using VICTOR® Nivo™ software.
Data analysis	Patch Clamp data was analyzed with Sophion Analyzer v6.6.70 (Sophion Bioscience) and GraphPad Prism v10.<br>BLI data was processed and analyzed using Octet® Analysis Studio 12.2.2.26 (ForteBio) and GraphPad Prism v10.<br>All other data analysis was performed on GraphPad Prism v10.

For manuscripts utilizing custom algorithms or software that are central to the research but not yet described in published literature, software must be made available to editors and reviewers. We strongly encourage code deposition in a community repository (e.g. GitHub). See the Nature Portfolio guidelines for submitting code & software for further information.

Data

Policy information about availability of data

All manuscripts must include a data availability statement. This statement should provide the following information, where applicable:

- Accession codes, unique identifiers, or web links for publicly available datasets
- A description of any restrictions on data availability
- For clinical datasets or third party data, please ensure that the statement adheres to our policy

All the data supporting the present manuscript is available in the form of Source Data Files and in the supplementary material. Relevant nanobody and toxin

sequences as well as detailed information on in vivo experiments are provided in the Supplementary Material. Raw data and analyses performed for the figures are available as Source Data Files.

Research involving human participants, their data, or biological material

Policy information about studies with human participants or human data. See also policy information about sex, gender (identity/presentation), and sexual orientation and race, ethnicity and racism.

Reporting on sex and gender	n/a
Reporting on race, ethnicity, or other socially relevant groupings	n/a
Population characteristics	n/a
Recruitment	n/a
Ethics oversight	n/a

Note that full information on the approval of the study protocol must also be provided in the manuscript.

Field-specific reporting

Please select the one below that is the best fit for your research. If you are not sure, read the appropriate sections before making your selection.

- [x] Life sciences
- [ ] Behavioural & social sciences
- [ ] Ecological, evolutionary & environmental sciences

For a reference copy of the document with all sections, see nature.com/documents/nr-reporting-summary-flat.pdf

Life sciences study design

All studies must disclose on these points even when the disclosure is negative.

Sample size	Samples sizes were selected according to standard protocols for each methodology. For in vivo assays, the smallest number of animals (3) that still render significant results was used. For electrophysiology, 16 individual cells per treatment were run in 384 well plates.
Data exclusions	No excluded data
Replication	Replicates were successful. For in vivo data, single experiments with 3 mice per group were done. All controls were done the same day. Enzymatic PLA2 assay replicates were perform as a single experiment in duplicates. Electrophysiology experiments were performed at the same time with cells patched individually in 384 well plates.
Randomization	Not relevant in lethality studies involving venom neutralization, as the outcome (death) is clearly evident
Blinding	Not relevant in lethality studies involving venom neutralization, as the outcome (death) is clearly evident

Reporting for specific materials, systems and methods

We require information from authors about some types of materials, experimental systems and methods used in many studies. Here, indicate whether each material, system or method listed is relevant to your study. If you are not sure if a list item applies to your research, read the appropriate section before selecting a response.

Materials & experimental systems

n/a	Involved in the study	
[ ]	[x]	Antibodies
[ ]	[x]	Eukaryotic cell lines
[x]	[ ]	Palaeontology and archaeology
[ ]	[x]	Animals and other organisms
[x]	[ ]	Clinical data
[x]	[ ]	Dual use research of concern
[x]	[ ]	Plants

Methods

n/a	Involved in the study	
[x]	[ ]	ChIP-seq
[x]	[ ]	Flow cytometry
[x]	[ ]	MRI-based neuroimaging

Antibodies

Antibodies used	The nanobody phage display library used in this study was prepared for the Center for Antibody Technologies from a llama and an

alpaca immunized with 18 elapid snake venoms at the VIB Nanobody Core (Brussels, Belgium).
The following commercial antibodies were used: HRP-conjugated anti-alpaca IgG VHH domain (128-035-232, Jackson ImmunoResearch) and anti-FLAG antibody clone M2 (F3165,Sigma-Aldrich).

Validation

From manufacturer's websites:
HRP-conjugated anti-alpaca IgG VHH domain:Alpaca Anti-Human IgG(Fcγ) HRP single domain Antibody. Recombinant Alpaca(VHH) Nano antibody.
Anti-FLAG M2: Monoclonal ANTI-FLAG® M2 is a purified immunoglobulin, IgG1, monoclonal antibody, isolated from murine ascites fluid, that binds to FLAG® fusion proteins. The M2 antibody will recognize the FLAG® sequence at the N-terminus, Met-N-terminus, C-terminus, or at an internal site of FLAG® fusion proteins. Monoclonal ANTI-FLAG® M2 is useful for identification and capture of FLAG® fusion proteins by common immunological procedures such as Western blots and immuno-precipitation. It is also useful for affinity purification of FLAG® fusion proteins when bound to a solid support. Monoclonal ANTI-FLAG® M2 binding is not dependent on calcium.

Eukaryotic cell lines

Policy information about cell lines and Sex and Gender in Research

Cell line source(s)

Gibco® CHO_S''' Cells: Gibco® CHO_S''' Cells (cGMP Banked) and Media Kit have been developed for the growth of Chinese Hamster Ovary (CHO) cells and expression of recombinant proteins in suspension culture. Parental CHO-S''' cells have been produced, banked and tested to meet current Good Manufacturing Practice regulations 21 CFR Parts 210,211,600 and 610. The cells have been adapted to CD CHO Medium for serum free suspension growth. The CD CHO Medium is made from animal origin free and chemically defined components. It contains no proteins, hydrolysates, or components of unknown composition. The medium is formulated without L-glutamine for greater stability, and without phenol red to minimize potential for estrogen-like effects.

Authentication

yes: cGMP Banked

Mycoplasma contamination

The cells are tested negative for mycoplasma contamination: cGMP Banked

Commonly misidentified lines
(See ICLAC register)

n/a

Animals and other research organisms

Policy information about studies involving animals; ARRIVE guidelines recommended for reporting animal research, and Sex and Gender in Research

Laboratory animals

Animal experiments were conducted in CD-1 mice of both sexes weighing 18-20 g (corresponding to 4-5 weeks old). Mice were supplied by the animal facility of the Instituto de Biotecnología, Universidad Nacional Autónoma de México (IBt-UNAM), Cuernavaca, Mexico. Mice were provided food and water ad libitum and housed in standard cages in groups of maximum 4 mice per cage. Animals were maintained at 18-24 °C, relative humidity of approximately 60% and 12:12 light-dark cycle.

Wild animals

An adult specimen of coral snake Micrurus diastema from Los Tuxtlas, Veracruz, Mexico was collected and kept for venom extraction at the "Herpetario Cantil" from the Instituto de Biotecnología, Universidad Nacional Autónoma de México (IBt-UNAM), Cuernavaca, Mexico (Collection license # SGPS/DGVS/03459/15, SEMARNAT, Mexico). The specimen was transported using a breathable cloth bag at temperatures between 25 and 30 °C and was kept in the herpetarium after the study for further research. All handling and housing procedures have been revised and accepted by the Mexican Ministry of Environment and Natural Resources (SEMARNAT) under the scientific collection agreement MOR-IN-166-0704.

Reporting on sex

Mice of both sexes weighing between 18 and 20 g. The sex of Micrurus diastema was not determined.

Field-collected samples

No field collected samples were used in this study

Ethics oversight

All in vivo methodologies used in the present work were previously approved by the bioethics committee of the Instituto de Biotecnologia,UNAM, Mexico, under project # 385 "Caracterización funcional y análisis de especificidad de venenos de coralillos Norteamericanos".

Note that full information on the approval of the study protocol must also be provided in the manuscript.

Plants

Seed stocks	n/a
Novel plant genotypes	n/a
Authentication	n/a
